# Genetic risk factors for periodontitis: a genome-wide association study using UK Biobank data

**DOI:** 10.1007/s00784-025-06205-8

**Published:** 2025-02-14

**Authors:** Chenyi Gao, Mark M. Iles, David Timothy Bishop, Harriet Larvin, David Bunce, Bei Wu, Huabin Luo, Luigi Nibali, Susan Pavitt, Jianhua Wu, Jing Kang

**Affiliations:** 1 Wolfson Institute of Population Health, Queen Mary University of London, London, UK; 2 School of Dentistry, University of Leeds, Leeds, UK; 3 Leeds Institute for Data Analytics, University of Leeds, Leeds, UK; 4 NIHR Leeds Biomedical Research Centre, Leeds Teaching Hospitals NHS Trust, Leeds, UK; 5 Leeds Institute of Medical Research, School of Medicine, University of Leeds, Leeds, UK; 6 School of Psychology, University of Leeds, Leeds, UK; 7 Rory Meyers College of Nursing, New York University, New York, US; 8 Department of Public Health, East Carolina University, Greenville, US; 9 Oral Clinical Research Unit, Faculty of Dentistry Oral Craniofacial Sciences, King’s College London, London, UK; 10 Periodontology Unit, Centre for Host Microbiome Interactions, Faculty of Dentistry, Oral & Craniofacial Sciences, King’s College, London, UK

**Keywords:** Periodontal disease, Genome, Genetic, GWAS

## Abstract

**Objectives:**

Periodontitis is linked with many health conditions, but its genetic basis is not yet understood. This genome-wide association study (GWAS) aimed to investigate the genetic variants associated with periodontitis.

**Materials and methods:**

This study utilised UK Biobank participants of European descent. Individuals were categorised as “having periodontitis” if they self-reported having ‘painful gums’, ‘bleeding gums’ or ‘loose teeth’ (n = 68,482), or as “controls” for those without these symptoms (n = 307,342). We conducted GWAS of this binary periodontitis phenotype using logistic regression models with PLINK2.0 adjusting for age, sex and the first 15 principal components to account for population stratification.

**Results:**

There were 376,611 participants (mean baseline age = 57 ± 7.9 SD) included in the GWAS, and four significant loci were identified: rs775476621 on chromosome 11 (Odds Ratio, OR[T]: 3.08, *p* = 1.01 × 10^− 8^), rs751014048 on chromosome 11 (OR[G]: 3.07, *p* = 1.04 × 10^− 8^), rs149922301 on chromosome 4 near gene *RP11-61G19.1 *(OR[A]: 1.18, *p* = 2.71 × 10^− 8^) and rs368467810 on chromosome 6 near gene *HIST1H3L* (OR[TTTA]: 0.96, *p* = 3.88 × 10^− 8^).

**Conclusions:**

Within the current limitations, such as self-reported phenotype and older age of the study population, four loci were detected for periodontitis that have not previously been linked with this condition. Further exploration of the function of these loci may contribute to improved understanding of periodontitis aetiology and subsequent drug development.

**Clinical relevance:**

These findings offer new targets for future research to investigate the genetic impact on periodontitis and aid the future understanding of periodontitis pathology and the disease’s progression.

**Supplementary Information:**

The online version contains supplementary material available at 10.1007/s00784-025-06205-8.

## Introduction

Periodontitis is a common inflammatory condition that affects the tissue surrounding and supporting the teeth [1]. Periodontitis is recognised as a major cause of tooth loss [1] and is associated with various long-term chronic conditions such as diabetes [2], cognitive impairment and dementia [3, 4], and cardiovascular disease [5], resulting in a worsening quality of life [6] and increased risk of mortality [7, 8]. Periodontitis affects 20–50% of the global population [9] with severe cases impacting more than 1 billion adults worldwide [10]. The pathology and aetiology of periodontitis are multifaceted, which includes the complex interplay of microorganisms, pathogens, environmental factors such as nutritional intake and smoking, and genetic factors [11–13]. While there have been numerous experimental studies into the role of microorganisms, pathogens and environmental factors in the development of periodontitis, there are also emerging studies exploring the genetic influences with 65 genes putatively associated with periodontitis [13].

Recent analyses have produced estimates of the heritability of periodontitis ranging from 7 to 38% [14]. To date, 16 genome-wide association studies (GWAS) of various forms of periodontitis have been conducted. 15 GWASs identified in the previous systematic review [15], and another GWAS of periodontitis published recently [16]. Here, 12 SNPs were collectively found that reached the conventional level for genome-wide statistical significance (*p* < 5 × 10^− 8^). However, no common loci have been identified across these studies, even amongst those of similar ethnicity [15]. This may be partly due to heterogeneity across studies and small sample size. Improved knowledge of the genetics of periodontitis could potentially benefit not only our understanding of disease aetiology, but also risk assessment and personalised treatment plans [17].

Further GWAS investigations using larger samples are warranted. The aim of this study, therefore, was to use data from the UK Biobank (UKB) to identify the genetic risk factors for periodontitis in a population of European descent.

## Method

### Sample and data resources

UKB [18] is a prospective cohort study of over 500,000 participants aged from 40 to 69 years at initial recruitment (2006–2010). A wide range of demographic and health-related variables and lifestyle data were recorded for participants through questionnaires, physical examination, imaging and genotyping at baseline. UKB continued to follow-up participants’ health-related outcomes through continued contact and access to medical records [19] (https://www.ukbiobank.ac.uk/).

In the current study, the sample was drawn from the final release of UKB genetic data (Sample size *n* = 488,377), out of which 409,548 (84% of total genotyped sample) persons have been classified as of European ancestry by integration of self-reported ethnicities and principal components analysis (PCA) results of population structure [18]. At the time of analysis, 139 UKB recruits had subsequently withdrawn their consent and so were excluded from this study.

The study report follows the STrengthening the REporting of Genetic Association Studies(STREGA)—An Extension of the STROBE Statement [20].

### Phenotype: periodontitis

The UKB variable “self-reported dental health” (variable code: 100538) was used as a surrogate for periodontitis. Participants who reported “Painful gums”, “bleeding gums”, or “loose teeth” were classified as having periodontitis; whereas, participants who reported other or no dental health issue were classified as control. Previous validation study suggested that self-reported measure of periodontitis has acceptable validity as surrogate for periodontitis [21]. Participants choosing “prefer not to say” were coded as missing for this variable. The same definition has been used in previous UKB studies [7, 22] in the absence of a clinical diagnosis.

### Genotyping and imputation

Detailed DNA extraction and quality filtering are described elsewhere (https://biobank.ctsu.ox.ac.uk/crystal/ukb/docs/ukb_dna_processing.pdf).

**Fig. 1:**
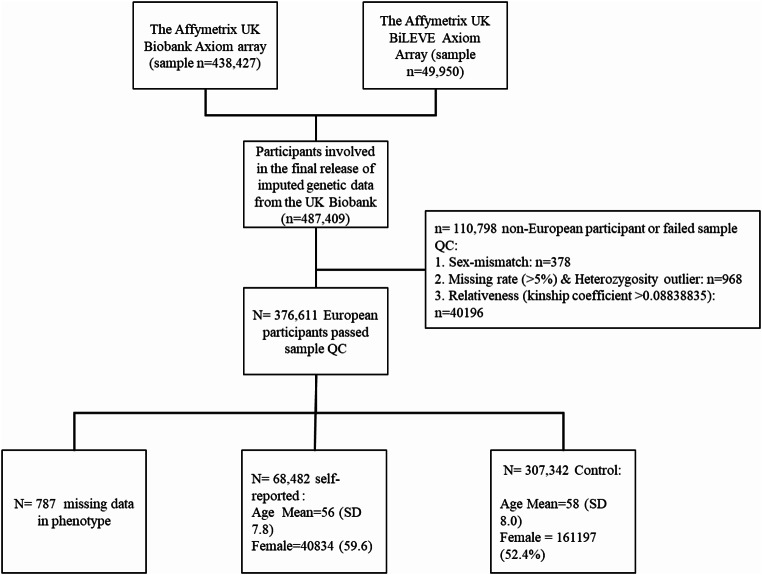
Flow chart showing the sample size selection by applying the quality control filter and ethnicity filter. Sample characteristics were also shown. SD, Standard Deviation; QC, quality control

Two genotyping arrays were employed: (1) the Affymetrix UK Biobank Axiom array and (2) the Affymetrix UK BiLEVE Axiom Array (Fig. 1). The genotyped samples shared 95% common single nucleotide polymorphisms (SNP) and produced 825,927 SNPs for genotyping in total. The imputation of missing genotypes was implemented in IMPUTE4 (https://jmarchini.org/software/), which was developed from IMPUTE2 undertaken by UKB using genotype reference data from the Haplotype Reference Consortium (HRC). The UK10K and 1000G genotype reference panels were employed to impute SNPs not included in HRC. This study performed quality control filtering and genome-wide analysis on Version 3 of the imputed data, containing 487,409 participants for 93,095,623 autosomal SNPs. Details of DNA extraction, genotyping and imputation are documented elsewhere [18].

UKB also provide the values of principal components (PC) for each participant to adjust for population stratification; as suggested by UKB, the first 15 are used in most analyses.

### Statistical analysis

Sample and variant exclusions were based on the quality control metrics provided by UKB. Only participants of European ancestry were included in this study. For sample quality control, UKB performed heterozygosity checks by fitting a linear regression model with the first six principal components and outlined 968 outliers with unusually high heterozygosity rate and missing call rate > 5%. The heterozygosity rate and missing call rate outliers were removed from the current study. Participants were also excluded for a discrepancy in biological sex and self-reported sex. Related pairs of participants were randomly removed one from every pair of participants with a kinship coefficient > 0.08838835 [23] which was calculated and provided by UKB. Based on the imputation information (INFO) scores and minor allele frequency (MAF) information provided by UKB, an exclusion list containing SNPs with INFO score ≤ 0.5 was created. Duplicated SNPs were also removed before GWA analyses, resulting in 58,443,190 included in the final GWAS.

GWA analyses were conducted on autosomal chromosomes using a logistic regression model that assumed an additive mode of inheritance, implemented in PLINK 2.0 (Linux) (https://www.cog-genomics.org/plink/2.0/) [24]. Two distinct association analyses were performed: (1) An association analysis without any covariates, and (2) An association analysis with age, sex and the first 15 principal components (PC1-15) included as covariates to adjust for baseline age, sex and population stratification. In the analysis, we defined the threshold for genome-wide significance as the conventional level of *p* < 5 × 10^− 8^ and a suggestive level of significance as *p* < 5 × 10^− 6^. Manhattan plots and Q-Q plots were created in R 4.2.2 (https://www.r-project.org/about.html), using the packages “qqman” [25] and “fastqq” (https://github.com/gumeo/fastqq). In addition, SNPs that were significant in previous GWASs reviewed by Gao, Iles [15] plus SNPs reported in [16] were also examined in our study to confirm replication of the results. The efficacy of genome-wide significant SNPs (*p* < 5 × 10^− 8^) in predicting periodontitis were evaluated using Receiver Operator Characteristic (ROC) curves [26] in the package “ROCR” in R4.2.2 [27].

### Functional annotation using FUMA 1.6.0

SNPs with MAF > = 0.001 from the model including covariates were followed up for functional annotation in FUMA v.1.6.1 [28] using function SNP2GENE. The default parameter in the SNP2GENE function was used with 1000 Genome phase 3 as the reference panel for its largest number of reference SNPs. SNPs with MAF > = 0.05 were entered into FUMA as sensitivity analysis.

SNPs were declared as independent significant loci if they reached *p* < 5 × 10^− 8^ and were in no more than weak linkage disequilibrium (r^2^ < 0.6). Lead SNPs at a locus were further defined as the most significant amongst those with r^2^ > 0.1. ANNOVAR (2017-07-17) was applied to estimate the functional consequence of SNPs. MAGMA (v1.08) was implemented to conduct gene-based tests, gene-set tests and gene-property tests with the Genotype-Tissue Expression (GTEx) v8 53 tissue types. Details of the function of FUMA and analysis of SNP2GENE are available in [28].

## Results

Following initial quality control, 378 sex mismatches between biological sex and self-reported sex, 968 participants with high SNP missing rates (> 5%) and unusually high heterozygosity rates as documented by UKB, plus 40,196 participants from related pairs were removed. Finally, 376,611 European participants (Mean age = 57.6 years old, 53.76% women, 18.2% periodontitis cases) remained, of which 787 participants were dropped from the association analysis due to missing phenotype data. The sample characteristics are presented in Fig. 1.

### SNPs—periodontitis association: null model and covariates model

**Fig. 2:**
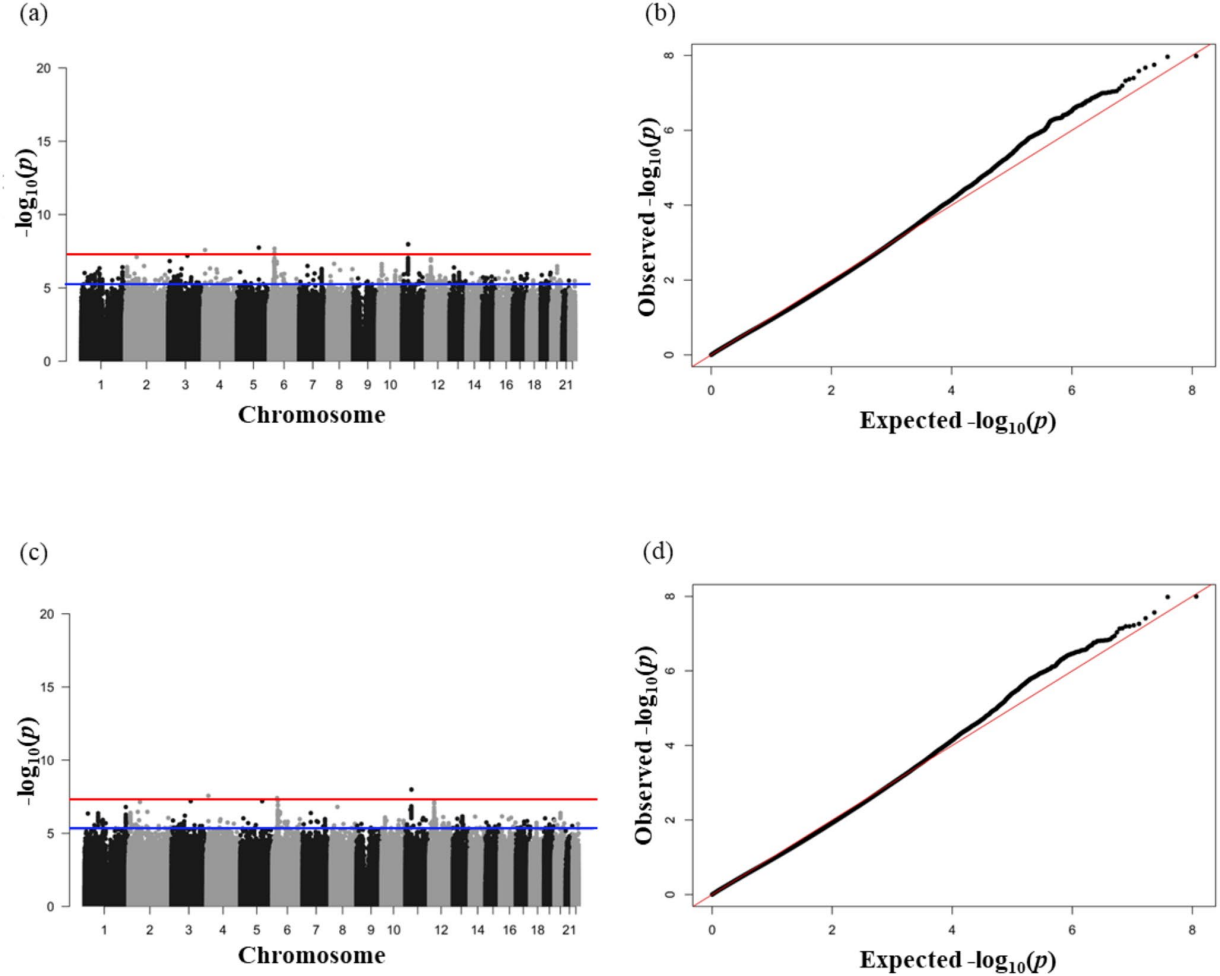
(**a**) Manhattan plot of the association analysis model without adjusting for any covariates, and (**b**) Corresponding Q-Q plot. (**c**) Association analysis model with adjustment for age, sex and first 1–15 PCs, and (**d**) Q-Q plots of corresponding model (GC lambda = 0.98). Note. The red line in (**a**) and (**c**) Manhattan plot represents the genome-wide significant threshold (*p* < 5 × 10^-8^); while the blue represents the suggestive line of significance (*p* < 5 × 10^-6^)

Manhattan plots are presented in Fig. 2 to show the association signals across the genome. In the null model (no covariates), 8 SNPs reached genome-wide significance (*p* < 5 × 10^− 8^) in relation to periodontitis (Table 1). After adjusting for 17 covariates (age, sex, PC1-15), three of the 8 SNPs remained genome-wide significant: rs775476621 (Odds Ratio_[T]_ = 3.08, *p* = 1.01 × 10^− 8^) and rs751014048 (OR_[G]_ = 3.07, *p* = 1.04 × 10^− 8^) on chromosome 11; rs149922301 (OR_[A]_ = 1.18, *p* = 2.71 × 10^− 8^) on chromosome 4. An additional SNP also reached significance in the covariates model: rs368467810 (OR_[TTTA]_ = 0.96, *p* = 3.88 × 10^− 8^) on chromosome 6. Overall, 674 SNPs (including four GWAS significant SNPs) reached our suggestive level of significance (*p* < 5 × 10^− 6^) in the covariates model, so may also contribute to periodontitis, and the results for these SNPs can be seen in Supplementary Table 1.

**Table 1:**
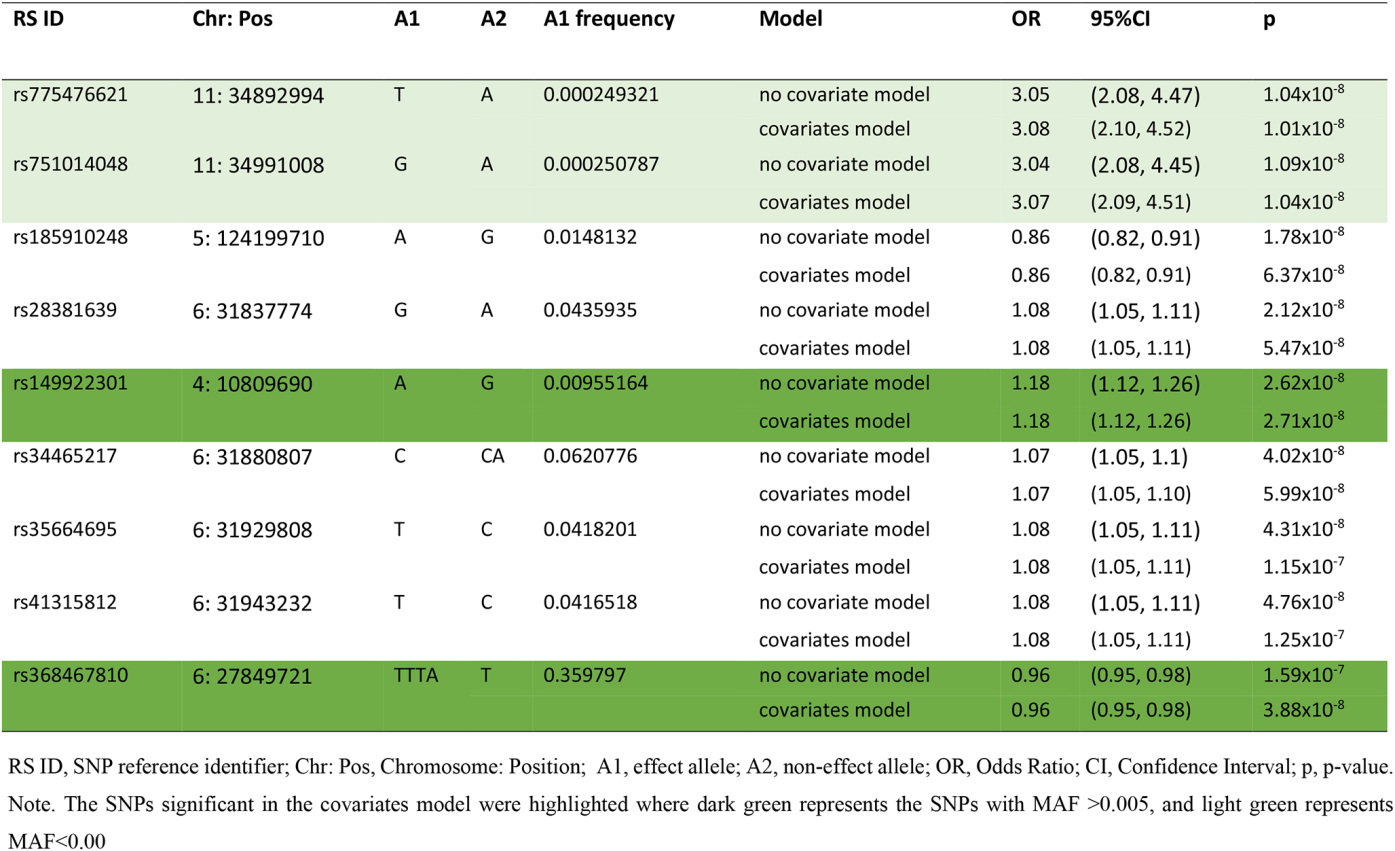
Association results for SNPs with *p* < 5 × 10^− 8^ for both models with and without covariates adjusted

QQ plots compared the observed p-value and expected p-value under the null hypothesis providing an indication of inflation in the GWAS results. A genomic inflation factor lambda = 0.98 for both models indicated no inflation in the results.

**Fig. 3:**
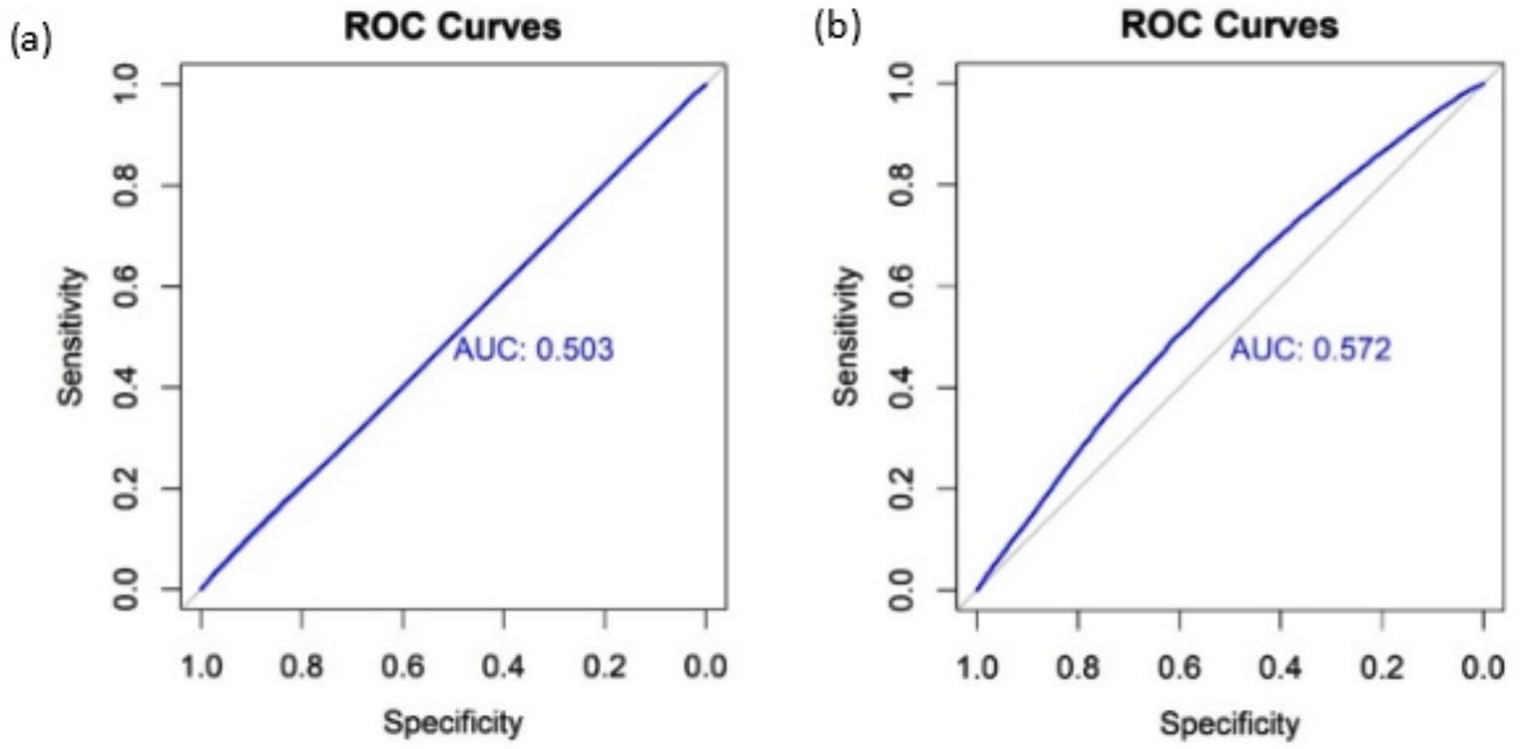
(**a**) ROC analysis including the significant SNPs from the association analysis model without adjustment for covariates. 2 (**b**) ROC analysis including the four significant SNPs from the association analysis model with adjustment for on age, sex, and first 1–15 principal components. The grey line represents the reference line (AUC = 0.500), and the area under curve (AUC) is marked along the reference line of both ROC curves

To cross-validate the accuracy and performance of our GWA models, significant SNPs from both models and covariates (age, sex, first 15 PCs) were entered into the ROC curve estimate, respectively. Better performance and model accuracy were observed for the covariates model (Area under curve (AUC) = 0.572) compared to the no covariates model (AUC = 0.503). Figure 3 shows the ROC curve with area under curve for both association models.

In addition to the results from the current study, SNPs reported as associated with periodontitis in 13 previous studies reviewed by Gao, Iles [15], were also investigated. Based on the results from our GWAS with covariates, none of the previously reported SNPs reached *p* < 5 × 10^− 8^ in our study (Supplementary Table 2).

### FUMA analysis

In the covariates model, there were 18,849,429 SNPs with (MAF < 0.001) imported to FUMA for further downstream functional analysis. Two leading and independent genome-wide significant SNPs (rs149922301, rs368467810) were identified, both located in intergenic regions, where 132 genes mapped onto leading SNPs, 31 of which are pseudogenes. Four genes were annotated to the two leading SNPs: (1) rs149922301: *RP11-61G19.1* (distance = 110405) and *MIR572* (distance = 560761); and (2) rs368467810: *HIST1H4L* (distance = 8432) and *HIST1H3L* (distance = 8472). The regional plot can be viewed in Supplementary Fig. 1.

In the gene set analysis, there were no genes found to be significant after Bonferroni correction, with *p =* 1 after Bonferroni correction for all gene sets (Supplementary Table 3). The MAGMA tissue expression analysis in this study found no significant association between gene expressed in all 53 tissue and periodontitis (Supplementary Fig. 2).

The results from the sensitivity analysis (MAF > = 0.05) also showed no significant association with all 53 tissue and any gene-sets. Results can be viewed in supplementary file.

## Discussion

The current study used UKB data to investigate the genetic risk variants for self-reported periodontitis and found four significant SNPs with two mapped onto gene *RP11-61G19.1* (rs149922301[A]), and *HIST1H3L* (rs368467810[TTTA]) associated with the self-reported periodontitis phenotype based on the fully adjusted model. The gene analysis of GWAS results prioritised 132 genes.

When compared to prior GWAS investigations of periodontitis, only two SNPs reported in previous studies reached nominal significance (*p* < 0.05/59 = 0.001) after Bonferroni correction in our results: rs12461706 [T] identified in Shungin, Haworth [17] (our study *p* = 5.34 × 10^− 4^), and rs11084095 [A] identified in Munz, Richter [29] (*p* = 4.58 × 10^− 4^) (Supplementary Table 2). None of the genome-wide significant (*p* < 5 × 10^− 8^) SNPs in our study were previously associated with periodontitis. Additionally, most of the previously reported SNPs did not achieve statistical significance (*p* < 0.05) in our GWAS.

There are several reasons for the lack of significance of the previously reported SNPs and why the SNPs highlighted here have not been reported before. One reason is the differences in the ethnic groups studied. For example, SNPs (rs2392520[C] *p* = 4.17 × 10^− 6^) discovered in Japanese sample [30] were not statistically significant (*p* < 0.05) in our sample (*p* = 0.52). Similarly, most of the genome-wide significant (*p* < 5 × 10^− 8^) and suggestive significant SNPs (*p* < 5 × 10^− 6^) discovered in Korean sample from Hong, Shin [31] were not significant in this study. The two SNPs reached nominal significance both included European sample [17, 29], although there is sample overlap between current study and Shungin, Haworth [17].

A second reason is the limited sample size in most prior studies, which may not be sufficient for a GWAS to find “true risk/protective SNPs”. For example, Petty, Silva [32] included only 879 mixed ethnicity participants (333 periodontitis cases) and did not find any genome-wide significant SNPs. Their suggestive SNPs (rs12800372 [C], *p* < 5 × 10^− 6^) did not reach statistical significance in our study. The two SNPs reached the nominal significance in the current study were from two large scale studies using European sample [17, 29], although the genome-wide significant SNP (rs12461706 [T]) from Shungin, Haworth [17] cannot be considered as replication due to overlapped sample used.

The third reason for the previously reported SNPs not reaching the same level of significance or nonsignificant in this study might be due to phenotype definition differences. For Shungin, Haworth [17], Munz, Richter [29] and the present study, although we all included European samples and had relatively larger sample sizes, different measures were used for periodontitis and varying case definitions employed. Shungin, Haworth [17] used mixed periodontitis measures and definitions as multiple datasets were included. Periodontitis measures included self-reported periodontitis, clinically diagnosed periodontitis and clinical examination measures, defined using criteria such as the CDC-AAP definition [33] and the 1999 international workshop for classification of periodontal diseases [34]. Munz, Richter [29] used mixed definitions as well which included the CDC-AAP definition [33] and definition based on bone loss ( > = 30%) or loss of attachment ( > = 4 mm) solely. Our study utilised a self-reported periodontitis definition encompassing bleeding gums, painful gums and loose teeth. This definition varied from Shungin, Haworth [17] who identified UKB participants with tooth loss only as a surrogate for periodontitis. These differences in case definitions are likely to have affected case and control classification in the final study population, thereby impacting the results.

Despite the differences, the significance (*p* < 10^− 3^) observed on the rs12461706 [T] locus from Shungin, Haworth [17], and rs11084095 [A] from Munz, Richter [29] partially support the important role of gene *SIGLEC5*. Both rs12461706 [T] and rs11084095 [A] were annotated on *SIGLEC5* which functioning innate immune systems and contributes to periodontitis [35].

The function of the four SNPs (i.e., rs775476621, rs751014048, rs149922301, rs368467810) identified in this study are not fully understood, especially SNPs rs775476621 and rs751014048 which had no functionality associated with them using FUMA. There are four genes annotated for the other two identified SNPs: (1) rs149922301: RP11-61G19.1 (distance = 110405) and *Micro RNA 572 (MIR572)* (distance = 560761), and (2) rs368467810: *HIST1H4L* (distance = 8432) and* HIST1H3L* (distance = 8472). Gene *MIR572* is attributed to microRNA class which are short non-coding regulatory RNAs involved in the expression of more than 60% of human genes [36, 37]. Previous studies have found *MIR572* is upregulated in several types of cancer/malignancy such as non-small cell lung cancer [38], ovarian cancer [39], renal cell carcinoma [40], contributing to malignant development, poor prognosis, and shortened survival time. However, the regulatory role of *MIR572* in periodontitis is not clear and requires further investigation to establish how changes in expression of *MIR572* is related to periodontitis progression.

In addition to *MIR572, HIST1H4L* and *HIST1H3L* are histone genes within the family of protein coding genes for regulating the DNA binding. Histone modification is essential to many biological processes including brain development and mental illness [34] such as schizophrenia, bipolar disorder, autistic spectrum disorder and depression [41]. This corresponds to the previous findings on associations between periodontitis and mental illness [22], and raises the possibility that the mental illness and periodontitis share similar pathological pathways. However, further exploration of the exact biological impact these genes have on periodontitis development is still necessary and especially, little is known about the function of *RP11-61G19.1*. Future study of the function of these four genes could improve understanding of the pathology and underlying mechanisms of periodontitis.

According to the ROC curve, both of our association analysis models provided some prediction. These results suggest the discovered eight SNPs in null models and four SNPs together with covariates (i.e., age, sex and PC1-15) provide modest to poor performance on predicting periodontitis. The no covariate model has AUC = 0.503 and covariate model has AUC = 0.572 respectively. The possibility of low to moderate prediction and performance is that periodontitis is a multi-facetted disease involving environmental factors, microorganisms and genetic influences. The improvement in model prediction and performance by adding covariates (i.e., age, sex, PC1-15) again also suggests the importance of non-genetic factors in periodontitis.

The current study has several strengths. First, the use of high-quality genetic data with standardised quality control increases the reliability of the results. The sample size was larger than most previous GWAS investigations into periodontitis and drew from an ethnically homogeneous population, to maximise power and minimise the likelihood of false positive results. Second, our functional analysis suggested some promising avenues for further exploration of the genetic causes of periodontitis.

This study also has some limitations. Firstly, participants with missing data were not utilised in the association analysis, and we cannot assess whether they are missing at random. Secondly, this study used self-reported oral health status as a proxy for periodontitis, which is not as ideal as clinical measures. Additionally, our results are based on population with European ancestry, not including ethnicities minorities. Another limitation of this study may impact on the significance of findings is that the current study included an older population where the impact of lifestyle, comorbidities or any other potential risk factors may contribute more than genetic factor, while for younger population genetic variants may play a much stronger role in periodontitis. Therefore, interpretation with caution is needed.

## Conclusion

The current study has identified four significant loci associated with periodontitis in a European population using a large high-quality dataset from the UKB. Future studies are needed to further explore the loci identified here to better understand the pathology of periodontitis.

## Electronic supplementary material

Below is the link to the electronic supplementary material.

Supplementary Material 1

Supplementary Material 2

Supplementary Material 3

## Data Availability

The SNPs reached suggestive significance level (5e-6) is provided within the manuscript or supplementary information files. UK Biobank data were used in the current study and application is required for access.
