# Correction to: Genetic risk factors for periodontitis: a genome-wide association study using UK Biobank data

**DOI:** 10.1007/s00784-025-06286-5

**Published:** 2025-03-20

**Authors:** Chenyi Gao, Mark M. Iles, David Timothy Bishop, Harriet Larvin, David Bunce, Bei Wu, Huabin Luo, Luigi Nibali, Susan Pavitt, Jianhua Wu, Jing Kang

**Affiliations:** 1 Wolfson Institute of Population Health, Queen Mary University of London, London, UK; 2 School of Dentistry, University of Leeds, Leeds, UK; 3 Leeds Institute for Data Analytics, University of Leeds, Leeds, UK; 4 NIHR Leeds Biomedical Research Centre, Leeds Teaching Hospitals NHS Trust, Leeds, UK; 5 Leeds Institute of Medical Research, School of Medicine, University of Leeds, Leeds, UK; 6 School of Psychology, University of Leeds, Leeds, UK; 7 Rory Meyers College of Nursing, New York University, New York, US; 8 Department of Public Health, East Carolina University, Greenville, US; 9 Oral Clinical Research Unit, Faculty of Dentistry Oral Craniofacial Sciences, King’s College London, London, UK; 10 Periodontology Unit, Centre for Host Microbiome Interactions, Faculty of Dentistry, Oral & Craniofacial Sciences, King’s College, London, UK

**Correction to: Clinical Oral Investigations**

10.1007/s00784-025-06205-8

The sentence is needed to be added in acknowledgment section: “***This research has been conducted using the UK Biobank Resource under Application Number: 54633***”.

This additional statement is required by the data resource and does not affect the interpretation of the findings.

The original article has been corrected.

