# Supplementary material for: Genetic risk factors for periodontitis: a genome-wide association study using UK Biobank data
Source: Clin Oral Investig. 2025 Feb 14;29(2):129. doi: 10.1007/s00784-025-06205-8 (PMC11828758; doi:10.1007/s00784-025-06205-8)
Supplement: Supplementary file 3 — Supplementary Material 3 [file 784_2025_6205_MOESM3_ESM.docx]

**Supplementary information**

Ethical approval and UKBB approval reference

UK Biobank study is ethnically approved by the North West Multicentre Research Ethnic Committee, UK (REF: 16/NW/0274). Participants were recruited prior to the data collect, and participants are free to withdraw their data at any time point of the study. This current study was provided access to the relevant data (UKBB approval reference: 54633).

Supplementary figure

Figure S1. the regional plot of the leading significant SNPs

1. rs149922301


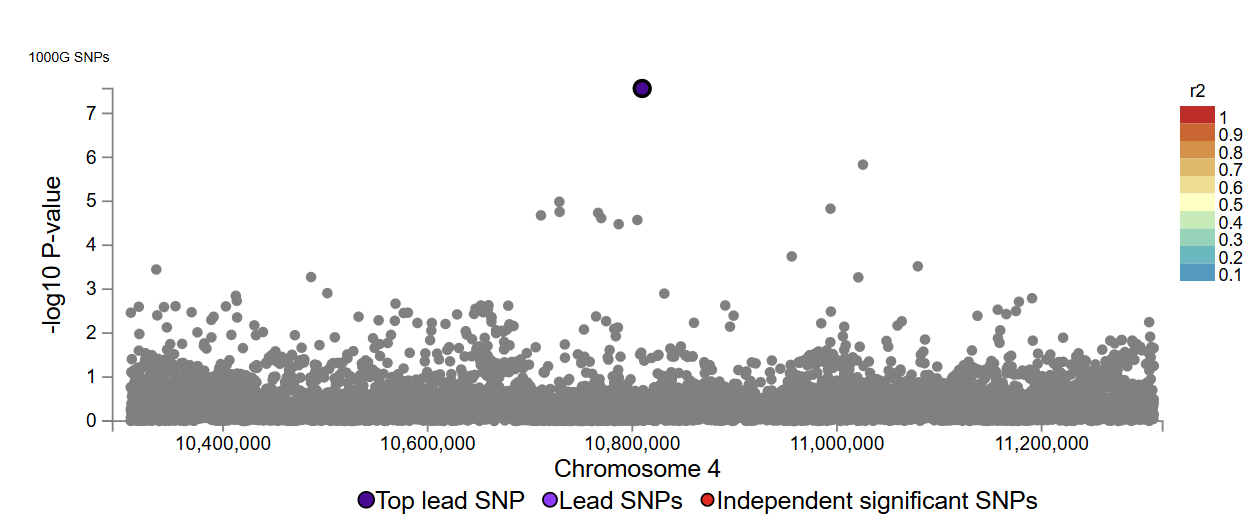


1. rs368467810


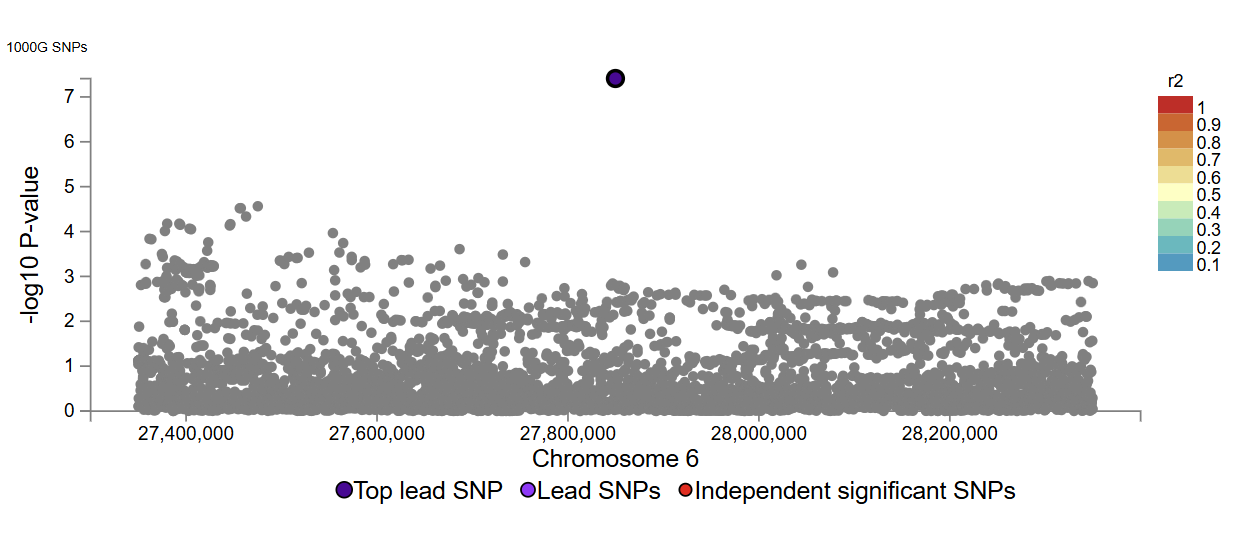


Figure S2. MAGMA tissue expression analysis using the Genotype-Tissue Expression (GTEx) v8 53 tissue types.

**
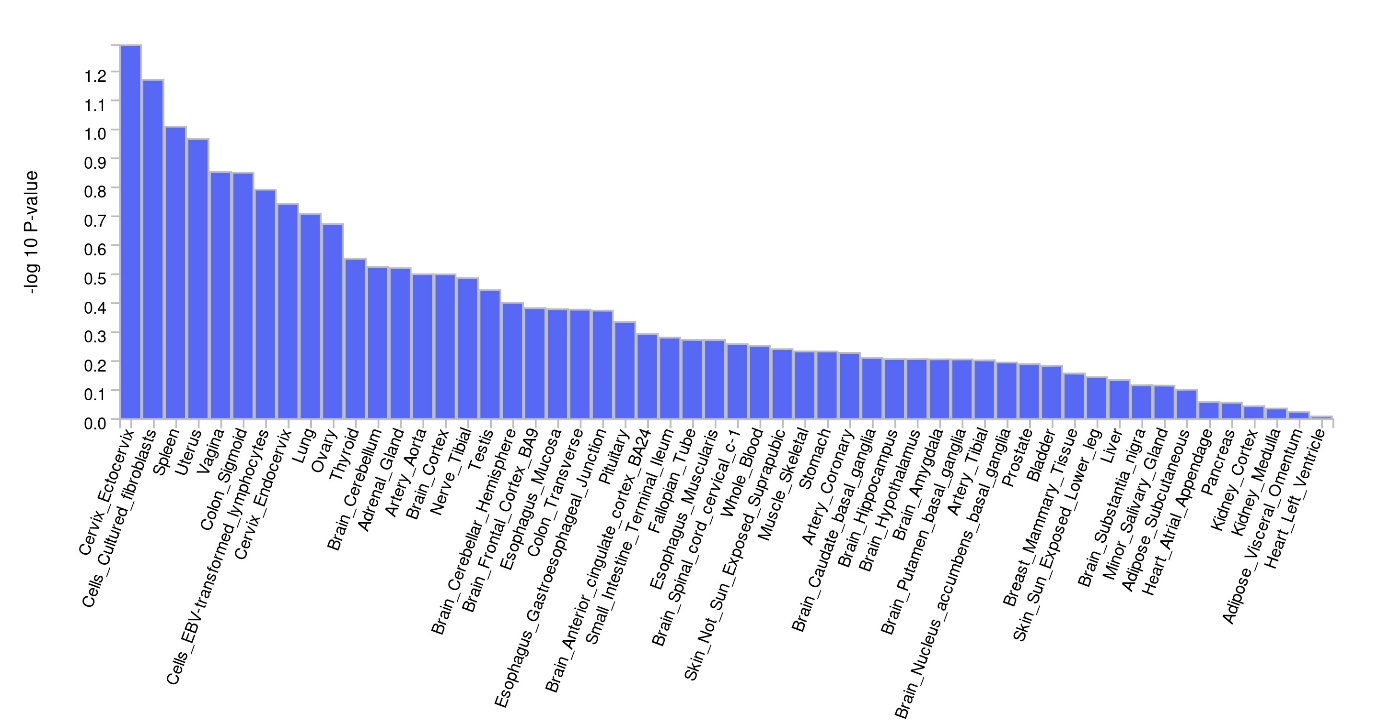
**

Note. The dash line represents the significant threshold and red bar represents tissue significantly associated with periodontitis. Missing dash line and red bar indicates there is no significant tissue expression found in MAGMA tissue expression analysis associated with periodontitis.

Table S1. Association results of SNPs (n=674) reach suggestive level of significance (p<5e-6) in the model adjusted for covariates.

(available in excel file “supplementary table 1”)

Table S2. Previous reported results and current GWAS results of previously reported significant SNPs (n=59).

|  | Previously reported significant SNPs | | | | association results in current study | | |
| --- | --- | --- | --- | --- | --- | --- | --- |
| Study | SNP [EA] | Nearest Gene | OR (95%CI) | P | EA | OR (95% CI) | p |
| Bevilacqua et al. (2017) | rs242016 [A] | CRACR2A | 3.7 (2.32, 6.29) | 1.50E-08 | A | 1.00 (0.99, 1.02) | 0.97 |
|  | rs242014 [T] | CRACR2A | 3.7 (2.32, 6.29) | 1.60E-08 | T | 1.00 (0.98, 1.02) | 0.99 |
|  | rs10491972 [G] | CRACR2A | 3.7 (2.32, 6.29) | 1.70E-08 | G | 1.00 (0.98, 1.02) | 1 |
|  | rs242002 [T] | CRACR2A | 3.6 (2.28, 6.13) | 2.73E-08 | T | 1.00 (0.98, 1.01) | 0.95 |
| deCoo et al. (2021) | rs35709256 [A] | FAT3 | 2.07 (1.55, 2.77) | 9.48E-07 | A | 1.00 (0.98, 1.02) | 0.87 |
|  | rs4807188 [A] | CSNK1G2 | 2.8 (1.83, 4.27) | 1.81E-06 | A | 0.95 (0.90, 1.00) | 0.047 |
|  | rs2074872 [A] | MYH13, LOC107985004 | 2.12 (1.55, 2.91) | 2.84E-06 | A | 1.00 (0.99, 1.01) | 0.92 |
|  | rs116611488 [T] | none | 4.22 (2.31, 7.72) | 2.90E-06 | T | 0.98 (0.93, 1.04) | 0.58 |
|  | rs4854545 [G] | ANTXR1 | 4.34 (2.34, 8.02) | 2.97E-06 | T | 0.99 (0.95, 1.03) | 0.47 |
|  | rs78672540 [C] | **none** | 2.32 (1.62, 3.31) | 3.78E-06 | C | 0.99 (0.96, 1.01) | 0.16 |
|  | rs13439823 [A] | ANGPT1 | 2.01 (1.49, 2.7) | 4.22E-06 | A | 1.00 (0.99, 1.02) | 0.5 |
|  | rs11993287 [A] | none | 0.58 (0.46, 0.73) | 4.31E-06 | A | 1.00 (0.99, 1.01) | 0.98 |
| Divaris et al. (2013) | rs2521634 [G] | LOC107986777 | 1.49 (1.28, 1.73) | 3.50E-07 | A | 1.00 (0.99, 1.01) | 0.95 |
|  | rs7762544 [G] | none | 1.4 (1.24, 1.59) | 7.50E-08 | A | 0.99 (0.98, 1.01) | 0.26 |
|  | rs3826782 [A] | ADGRE1 | 2.01 (1.52, 2.65) | 8.20E-07 | A | 1.01 (0.99, 1.04) | 0.27 |
| Hong et al. (2015) | rs4242220 [MA: C] | TENM2 | 0.53 (0.41, 0.69) | 2.84E-06 | G | 1.00 (0.99, 1.01) | 0.95 |
|  | rs12969041 [MA: A] | none | 2.86 (1.92, 4.27) | 2.79E-07 | T | 1.00 (0.98, 1.01) | 0.56 |
|  | rs2027756 [MA: A] | none | 2.86 (1.92, 4.27) | 2.79E-07 | T | 1.00 (0.98, 1.01) | 0.55 |
| Munz et al. (2017) | rs2978951 [A] | none | 1.25 (1.16, 1.35) | 2.06E-08 | G | 1.01 (1.00, 1.02) | 0.21 |
|  | rs2738058 [T] | none | 1.28 (1.18, 1.38) | 6.78E-10 | C | 1.02 (1.00, 1.03) | 0.01 |
|  | rs4284742 [G] | SIGLEC5 | 1.34 (1.21, 1.48) | 1.34E-08 | G | 0.99 (0.97, 1.00) | 0.08 |
|  | rs4970469 [G] | none | 1.52 (1.29, 1.81) | 1.20E-06 | A | 1.01 (0.99, 1.03) | 0.42 |
|  | rs1122900 [A] | none | 1.27 (1.16, 1.4) | 8.00E-07 | C | 0.99 (0.98, 1.01) | 0.3 |
|  | rs2070901 [T] | FCER1G | 1.29 (1.16, 1.44) | 4.36E-06 | T | 1.01 (1.00, 1.02) | 0.1 |
| Munz et al. (2019) | rs729876 [T] | LOC107984137 | 1.23 (1.15, 1.32) | 1.21E-08 | C | 1.01 (0.99, 1.02) | 0.44 |
|  | rs11084095 [A] | SIGLEC5 - AC018755.18 | 1.17 (1.11, 1.24) | 5.09E-08 | A | 1.02 (1.01, 1.03) | 4.58E-04 |
|  | rs9982623 [C] | MCM3AP | 1.23 (1.13, 1.33) | 8.65E-07 | T | 1.02 (1.00, 1.04) | 0.04 |
| Sanders et al. (2017) | rs149133391 [T] | TSNAX-DISC1 | beta: -0.139 (-0.09, -0.19) | 7.90E-09 | C | 1.08 (0.92, 1.27) | 0.33 |
|  | rs75715012 [G] | none | beta: 0.045 (0.03, 0.06) | 1.10E-07 | A | 0.99 (0.97, 1.01) | 0.38 |
|  | rs186066047 [G] | none | beta: 0.23 (0.14, 0.31) | 1.70E-07 | A | 0.34 (0.08, 1.47) | 0.15 |
|  | rs10456847 [C] | none | beta: -0.03(-0.04, -0.02) | 2.60E-07 | G | 0.99 (0.98, 1.00) | 0.06 |
|  | rs79308117 [A] | ASH1L | beta: 0.18 (0.11, 0.25) | 2.80E-07 | C | 0.55 (0.23, 1.33) | 0.19 |
| Schaefer et al. (2010) | rs1537415 [G] | GLT6D1 | 1.59 (1.36, 1.86) | 5.51E-09 | C | 1.02 (1.00,1.03) | 8.14E-03 |
| Shaffer et al. (2014) | rs733048 [not found] | LOC105374494 | 2.4 () | 1.00E-06 | A | 1.01 (1.00, 1.02) | 0.16 |
|  | rs10457525 [not found] | LOC102723409 | 2.33 () | 3.50E-06 | T | 1.00 (0.98, 1.01) | 0.66 |
|  | rs7749983 [not found] | LOC102723409 | 2.39 () | 2.40E-06 | A | 0.99 (0.98, 1.01) | 0.35 |
| Shimizu et al. (2015) | rs9446777 [A] | KCNQ5 | 0.86 (0.80, 0.93) | 4.83E-06 | G | 0.99 (0.96, 1.03) | 0.62 |
|  | rs2392510 [C] | GPR141 | 0.87 (0.82, 0.92) | 4.17E-06 | T | 1.00 (0.98, 1.01) | 0.52 |
| Shungin et al. (2019) | rs12461706 [T] | SIGLEC5 | 1.05 () | 3.90E-09 | T | 1.02 (1.01, 1.03) | 5.34E-04 |
| Tegelberg et al. (2021) | rs200392355 [CT] | LOC102724234 | beta: 0.16 (0.09, 0.22) | 1.22E-06 | NA | | |
|  | rs2409703 [C] | *XKR6* | beta: 0.28 (0.16, 0.39) | 1.61E-06 | C | 1.02 (1.00, 1.04) | 0.1 |
|  | rs11630851 [T] | DNM1P35 | beta: 0.30 (0.18, 0.42) | 9.39E-07 | T | 1.00 (0.97, 1.02) | 0.8 |
|  | rs4444613 [A] | TASP1 | beta: -0.28 (-0.38, -0.18) | 1.35E-07 | A | 0.99 (0.97, 1.01) | 0.26 |
|  | rs2003705 [T] | LOC107985448 | beta: -0.16 (-0.23, -0.10) | 1.68E-06 | T | 1.01 (1.00,1.02) | 0.21 |
| Petty et al., (2023) | rs12036106 [T] | RAP1GAP,USP48 |  | 5.07E-07 | T | 1.02 (1.00, 1.04) | 0.03 |
|  | rs13031512 [A] | TFPI,LINC01090 |  | 2.60E-06 | A | 0.99 (0.97, 1.00) | 0.15 |
|  | rs72870126 [G] | MEPE,SPP1 |  | 4.80E-06 | G | 0.99 (0.97, 1.02) | 0.55 |
|  | rs369717575 [TA] | PALLD |  | 1.96E-06 | NA | | |
|  | rs36793 [T] | LINC01848,TMEM232 |  | 2.07E-06 | T | 0.98 (0.93, 1.04) | 0.5 |
|  | rs148550758 [C] | LINC02487,LINC01558 |  | 2.04E-06 | C | 0.95 (0.89, 1.02) | 0.14 |
|  | rs7835237 [G] | STC1,ADAM28 |  | 3.62E-06 | G | 1.01 (0.97, 1.05) | 0.57 |
|  | rs12800372 [C] | TPCN2,LOC338694 |  | 4.34E-06 | C | 1.01 (1.00, 1.02) | 0.2 |
| Silvia et al. (2023) | rs2070902 [T] | *FCER1G* | 1.38 (1.24, 1.52) | 1.02E–09 | T | 1.01 (1, 1.03) | 0.08 |
|  | rs10988663 [T] | *HMNC2* | 3.32 (2.15, 5.14) | 6.07E–08 | T | 1.02 (0.97, 1.07) | 0.44 |
|  | rs150956098 [G] | *ZNF248* | 1.69 (1.35, 2.13) | 5.19E–06 | G | 1.02 (0.99, 1.05) | 0.31 |
|  | rs75527084 [C] | *LINC00355* | 0.44 (0.31, 0.62) | 3.20E–06 | T | 0.99 (0.94, 1.04) | 0.66 |
|  | rs7224672 [G] | *MIR4522* | 1.29 (1.16, 1.45) | 4.31E–06 | G | 1.01 (0.99, 1.02) | 0.35 |
|  | rs72832278 [A] | *GPR179* | 0.63 (0.52, 0.77) | 3.23E–06 | G | 1.01 (0.98, 1.04) | 0.52 |
|  | rs11084094 [C] | *SIGLEC5* | 1.27 (1.16, 1.38) | 1.36E–07 | C | 1.02 (1, 1.03) | 7.00E-03 |
| EA, effect allele; MA, minor allele; OR, Odds Ratio; CI, Confidence interval. Note. The beta marked in OR column means the results are from linear model and the results are corresponding coefficient beta and confidence interval. | | | | | | | |

Table S3. The top 10 results of Gene-set analysis

| **Gene Set** | **N genes** | **Beta** | **Beta STD** | **SE** | **P** |
| --- | --- | --- | --- | --- | --- |
| GOBP_REGULATION_OF_EPITHELIAL_CELL_DIFFERENTIATION | 148 | 0.26018 | 0.02277 | 0.075863 | 0.00030296 |
| HOWLIN_CITED1_TARGETS_1_UP | 33 | 0.54028 | 0.022395 | 0.15891 | 0.00033767 |
| WP_MELANOMA | 66 | 0.37145 | 0.021756 | 0.10986 | 0.00036184 |
| GOMF_CALCIUM_ACTIVATED_CATION_CHANNEL_ACTIVITY | 25 | 0.56123 | 0.020253 | 0.16671 | 0.00038154 |
| DAZARD_RESPONSE_TO_UV_NHEK_DN | 294 | 0.18309 | 0.022498 | 0.054564 | 0.00039681 |
| GOBP_CELLULAR_RESPONSE_TO_UV_B | 12 | 0.83387 | 0.020855 | 0.24917 | 0.00040985 |
| GOCC_DENDRITIC_TREE | 575 | 0.12584 | 0.021463 | 0.037756 | 0.0004305 |
| PHONG_TNF_RESPONSE_VIA_P38_COMPLETE | 216 | 0.19809 | 0.020907 | 0.059507 | 0.00043681 |
| GOBP_CELL_CYCLE_PROCESS | 1211 | 0.0818 | 0.019898 | 0.024652 | 0.00045382 |
| BOYAULT_LIVER_CANCER_SUBCLASS_G1_UP | 113 | 0.26765 | 0.020487 | 0.08073 | 0.00045846 |

P_bon,_ p value after Bonferroni correction; STD, standard deviation; SE, standard error.

Table S4. The top 10 results of MAGMA Gene-set analysis from sensitivity analysis (MAF>=0.05)

| **Gene Set** | **N genes** | **Beta** | **Beta STD** | **SE** | **P** | **P_bon_** |
| --- | --- | --- | --- | --- | --- | --- |
| HUMMERICH_BENIGN_SKIN_TUMOR_DN | 19 | 0.8755 | 0.027859 | 0.2134 | 2.0517e-05 | 0.348973653 |
| REACTOME_INITIATION_OF_NUCLEAR_ENVELOPE_NE_REFORMATION | 18 | 0.80493 | 0.02493 | 0.22008 | 0.00012779 | 1 |
| COLLER_MYC_TARGETS_DN | 6 | 1.5616 | 0.027934 | 0.4275 | 0.00013 | 1 |
| GOBP_LUNG_SACCULE_DEVELOPMENT | 9 | 1.0314 | 0.022593 | 0.28709 | 0.00016422 | 1 |
| GOBP_REGULATION_OF_RESPIRATORY_BURST | 15 | 0.8015 | 0.022663 | 0.22317 | 0.00016495 | 1 |
| GOBP_POSITIVE_REGULATION_OF_CELL_MATURATION | 7 | 1.4262 | 0.027554 | 0.40509 | 0.00021584 | 1 |
| GOBP_REGULATION_OF_MESENCHYMAL_STEM_CELL_DIFFERENTIATION | 8 | 1.2095 | 0.02498 | 0.34452 | 0.0002242 | 1 |
| GOBP_EPITHELIAL_CELL_DEVELOPMENT | 200 | 0.22434 | 0.023048 | 0.064102 | 0.00023352 | 1 |
| HOLLEMAN_DAUNORUBICIN_B_ALL_UP | 10 | 0.95749 | 0.022109 | 0.27379 | 0.00023575 | 1 |
| GOBP_POSITIVE_REGULATION_OF_TYPE_I_INTERFERON_PRODUCTION | 53 | 0.40408 | 0.021455 | 0.11744 | 0.00029079 | 1 |

P_bon,_ p value after Bonferroni correction; STD, standard deviation; SE, standard error.

Table S5. MAGMA tissue expression analysis using the Genotype-Tissue Expression (GTEx) v8 53 tissue types from sensitivity analysis (MAF>=0.05)
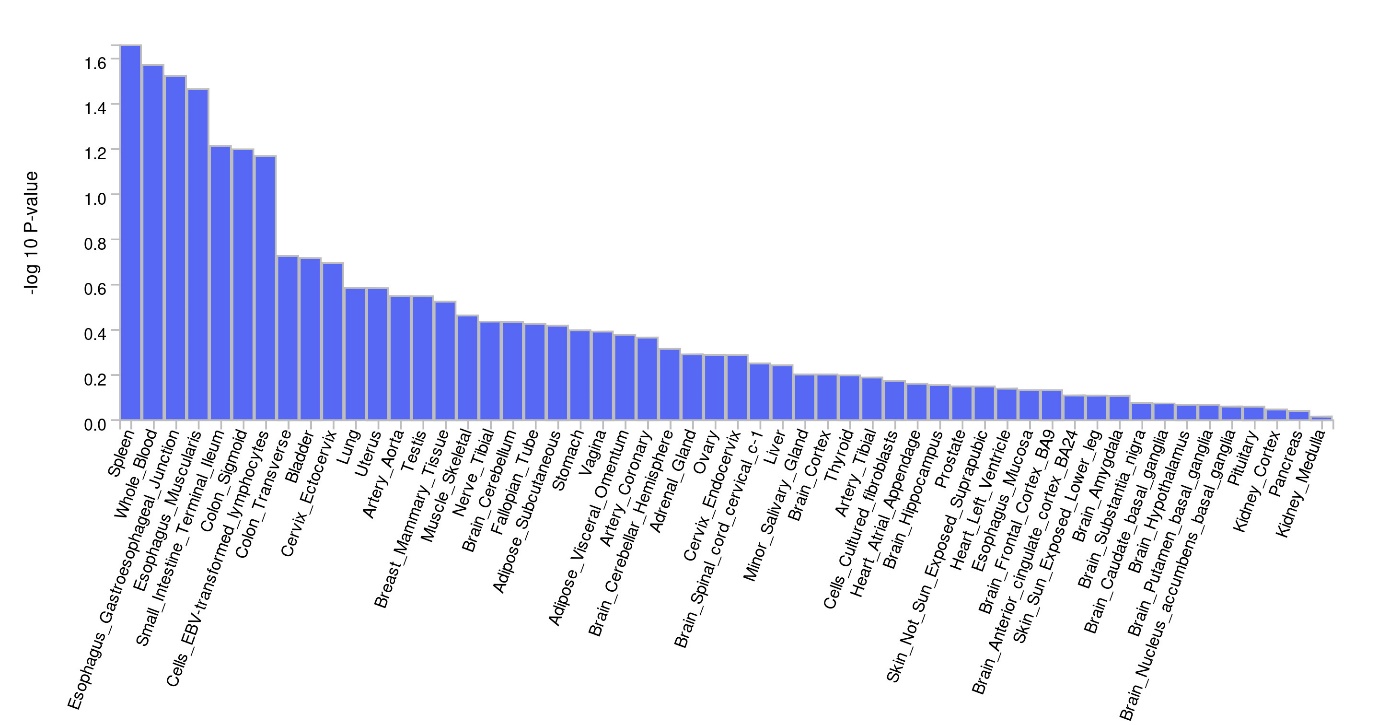


Note. The dash line represents the significant threshold and red bar represents tissue significantly associated with periodontitis. Missing dash line and red bar indicates there is no significant tissue expression found in MAGMA tissue expression analysis associated with periodontitis.
